# THM model of rock tunnels in cold regions and numerical simulation

**DOI:** 10.1038/s41598-024-53418-0

**Published:** 2024-02-12

**Authors:** Naifei Liu, Shihao Liang, Shuangjie Wang, Zhanping Song

**Affiliations:** 1 CCCC First Highway Consultants CO., LTD., State Key Laboratory of Road Engineering Safety and Health in Cold and High-Altitude Regions, Xi’an, 710075 China; 2 School of Civil Engineering, Xi’an University of Architecture and Technology, Xi’an, 710055 China; 3 Shaanxi Key Laboratory of Geotechnical and Underground Space Engineering, XAUAT, Xi’an, 710055 China

**Keywords:** Engineering, Civil engineering

## Abstract

The freezing damage of rock tunnels in cold region involves ice-water phase change and complicated interaction of Thermo–Hydro–Mechanical (THM) field. Taking the fractured rock mass of cold region tunnels as research subject, the THM coupling model of cold region tunnels was established, which is based on the seepage mechanics, heat transfer theory, damage mechanics and equivalent continuum theory. This model could reflect the anisotropic properties of deformation, water migration and heat transfer caused by the initial fracture of rock mass. The construction and operation processes of a rock tunnel in cold region were simulated, and results were compared with the measured value and predecessor’s achievements. It shows that proposed model could reflect the anisotropic property of surrounding rock and the simulated deformation and stress are not symmetrical. Compared with the literature, the calculated results in this paper are closer to the measured values. The insulating layer has a significant effect on the stress of the supporting structures. The maximum tension stress of the lining is 4.5 times as that without insulating layer, and the lining will be destroyed for the overlarge tension stress.

## Introduction

According to statistics, frozen soil regions encompass approximately 50% of the Earth's total land area and are predominantly distributed in polar and nearby areas as well as high-altitude regions^1^. The cold climate and frozen terrain not only create awe-inspiring landscapes, but also pose challenges for engineering construction and resource extraction. As the third largest permafrost country of the world, China possesses extensive cold regions in the northeast, western mountains, and plateau areas. Due to the unique properties of frozen rock mass, freezing damage often occurs in the construction and operation of projects in cold regions. There is even a saying that "for every ten tunnels in cold regions, nine is affected by freezing damage". The Yuximolegai Tunnel on National Highway 217 in Xinjiang was decommissioned due to frost damage. Similarly, the Guanjiao Tunnel at the Xining-Golmud section of the Qinghai-Tibet Railway undergoes heaving during winter and mud eruption during summer^2^. In fact, it is not only China, according to statistics, 1100 of the 3800 railway tunnels in Japan endanger driving safety during winter operation because of freezing damage, and tunnels in cold areas in Norway also have varying degrees of freezing damage. The surrounding rock of the above tunnels is mostly rock mass, so it is urgent to carry out the research of rock mass tunnel in cold area, especially the Thermo–Hydro–Mechanical (THM) coupling process.

The most notable distinction between tunnels in cold regions and those at normal temperatures is the presence of an ice-water phase transition induced by low temperatures. Consequently, research on tunnels in cold regions has been conducted by scholars both domestically and internationally from multiple perspectives, including laboratory tests, field monitoring, theoretical analysis, and numerical simulation, in conjunction with relevant tunnel projects in cold regions. The achievements of Chinese geotechnical engineers in cold region tunnel projects, particularly in conjunction with major infrastructure developments such as the Qinghai-Tibet Highway and Qinghai-Tibet Railway, have significantly facilitated the construction of cold region engineering in China. As early as 1999, a mathematical model addressing the coupling problem of temperature fields, seepage fields, and stress fields in tunnels with phase change in cold areas was proposed by Lai et al.^3,4^. They derived its finite element format using the Galerkin method and obtained frost heave forces and lining stresses in cold area tunnels. Xu^5^ and Tan^6^ derived the Thermo–Hydro–Mechanical (THM) coupling model of rock mass under low-temperature freeze–thaw conditions based on continuum mechanics theory. Zhang et al.^7,8^ established a three-dimensional calculation model for the coupling problem of temperature fields and seepage fields in cold region tunnels and conducted simulation analyses of the Fenghushan tunnel and Kunlunshan tunnel on the Qinghai-Tibet railway, highlighting the obstructive role played by insulation materials in refreezing. Yang et al.^9^ employed Femlab software to numerically simulate the temperature fields and water fields of surrounding rock in the exit section of the Osakashan tunnel in cold areas, analyzing the law of coupled hydrothermal migration in the soft rock tunnel. Jiang et al.^10^ proposed two air supply modes for maintaining the air temperature in cold area tunnels during the construction process: a heating preheating ventilation system and an ordinary ventilation system. Takumi^11^ and Han^12^ deduced analytical solutions for the tunnel temperature field and frost heave load, respectively. Deng et al.^13^ introduced the constrained frost heave model of a tunnel in cold regions, with frost heave pressure analogous to gas pressure. Qiu et al.^14^ conducted indoor frost heave tests on different tunnel section types and analyzed the distribution of frost heave forces in lining. Chen et al.^15^ proposed the idea of establishing a temperature-percolation-stress-damage coupling model for tunnels in cold areas based on the study of thermodynamic parameters and a multitude of freeze–thaw and compression tests. Kang et al.^16^ investigated the frost heave deformation characteristics of complete rock blocks through laboratory tests and simulated and analyzed the deformation characteristics of tunnels in cold areas. Xia et al.^17^ simplified the distribution pattern of rock fissures as "layered" and derived the formula for rock frost heave rate. Song et al.^18^ introduced a remote real-time monitoring system for the freeze–thaw cycles of plateau-frozen soil tunnels. Qu et al.^19^ studied the frost heave force induced by water migration in cracks through laboratory experiments. Feng et al.^20^ proposed an analytical algorithm for melt analysis of tunnels' surrounding rock in seasonal cold areas. Yuan et al.^21^ suggested criteria for distinguishing between freeze–thaw and freezing environments in tunnels' surrounding rock in cold areas. Li et al.^22^ emphasized that active thermal protection of shallowly buried sections of tunnels in permafrost areas, through the use of a hot rod group, can rapidly eliminate thermal interference caused by construction to the frozen surrounding rock of the tunnel. Zhang et al.^23^ established the frost heave model of crushing rings and deduced the formula for frost heave force based on displacement continuity conditions. A new frost heave model^24^ was subsequently established. Zheng et al.^25^ delved into the mechanism of lining cracking at tunnel entrances during winter and water leakage during the spring thaw period. In conclusion, current research on cold region tunnels primarily focuses on soil tunnels, with only a few studies considering rock tunnels, which are still assumed to be porous and continuous media. By adopting the theory of continuum mechanics to construct a corresponding mathematical model, the most significant characteristic of rock mass, that is anisotropy, is overlooked, making it difficult to accurately analyze practical problems related to rock tunnels in cold regions.

Anisotropy characteristics represent the fundamental attributes of engineering rock mass, while the ice-water phase transition constitutes the principal disparity between frozen tunnel and normal tunnel. In this paper, an anisotropic THM coupling model of rock tunnel in cold region will be constructed by taking equivalent continuously fractured rock mass as the research object. The THM model is employed for the numerical simulation of the entrance section of the Kunlunshan tunnel on the Qinghai-Tibet railway. It is studied the influence of insulation layer on deformation of surrounding rock and the stress of the supporting structures under different working conditions such as construction and operation period. The research results can provide reference for the design and construction of similar projects.

## The THM model of a rock mass tunnel in cold regions

The primary distinction between the THM coupling in frozen rock tunnels and their counterparts in normal rock tunnels lies in the involvement of the ice-water phase transition. Similarly, the principal contrast in THM coupling for frozen soil tunnels is the anisotropy stemming from fractures. Therefore, this study aims to derive the stress equilibrium equation, continuity equation, and energy conservation equation for rock tunnels in cold regions. Subsequently, a THM coupling model for rock tunnels in cold regions is formulated.

### Basic assumptions

In addressing the THM coupling problem within rock tunnels located in cold regions, this study establishes the following fundamental assumptions while adequately reflecting the primary contradictions:

1. The subject of investigation comprises fractured rock masses amenable to equivalent continuous treatment.
2. Mechanical responses are governed by the hypothesis of small deformation.
3. Each component within the characterization unit maintains a uniform temperature, consistent with the principles of mixture theory.
4. Heat conduction within each component follows Fourier's law.
5. The phase transformation process of ice-water is described using the Clausius–Clapeyron equation.

### Stress equilibrium equations

The momentum balance equation can be derived from the momentum balance principle. After sorting, the static balance equation in the form of effective stress can be obtained as follows^26^:

1 $$\left[ {\sigma^{\prime}_{ij} + \left( {\alpha_{w} p_{w} + \alpha_{i} p_{i} } \right)\delta_{ij} } \right]_{,j} + \rho_{e} {\mathop{X}\limits^{\rightharpoonup}} _{i} = 0$$

where σ′_*ij*_ is the effective stress tensor, *P*_*w*_ is the water pressure, *P*_*i*_ is the ice pressure, *α*_*w*_ and *α*_*i*_ are the effective stress coefficient (related to the initial damage tensor caused by the fracture), *δ*_*ij*_ is the Kronecker symbol, *ρ*_*e*_ is the density of the Representative Volume Element (RVE) of the fractured rock mass,${\mathop{X}\limits^{\rightharpoonup}}_{i}$ = (0,0,*g*)^*T*^ is the volume force when only gravity is considered.

Assuming that the thermal strain caused by temperature change is independent of other mechanical strains, the total elastic strain should include two parts: mechanical strain and temperature strain. Then the strain of the representative volume element of the rock tunnel surrounding rock in cold areas can be expressed as^27^:

2 $$\varepsilon_{ij} - \varepsilon_{ij}^{T} = \left( {C_{ijkl}^{0} + C_{ijkl}^{d} } \right)\sigma^{\prime}_{kl}$$

where, $\varepsilon_{ij}^{{}}$ is the total strain of fractured rock mass; $\varepsilon_{ij}^{T}$ is the thermal strain of the fractured rock mass. $C_{ijkl}^{0}$ is the elastic flexibility tensor of the rock matrix; $C_{ijkl}^{d}$ is the additional flexibility tensor due to the presence of cracks.

To arrange Eq. (2) to be an effective stress expression expressed by strain, we can get:

3 $$\begin{aligned} \sigma^{\prime}_{ij} & = \left( {C_{ijkl}^{0} + C_{ijkl}^{d} } \right)^{ - 1} \left( {\varepsilon_{kl} - \varepsilon_{kl}^{T} } \right) \\ & = K_{ijkl} \left[ {\varepsilon_{kl} - \beta_{r} \left( {T_{r} - T_{r0} } \right)\delta_{kl} } \right] \\ \end{aligned}$$

where *K*_*ijkl*_ is the initial stiffness tensor of the fractured rock mass, *C*_*ijkl*_ = $C_{ijkl}^{0}$ + $C_{ijkl}^{d}$ is the inverse tensor of the initial flexibility tensor, *ε*_*kl*_ is the total strain of the tunnel rock mass in the cold area, *β*_*r*_ is the thermal expansion coefficient of the rock matrix, *T*_*r*_ is the temperature of the rock matrix and *T*_*r*0_ is the reference temperature of the rock matrix.

By bringing Eq. (3) into Eq. (1), the anisotropic stress balance equation of a rock tunnel in cold areas can be obtained considering the thermoelastic influence:

4 $$\left\{ {K_{ijkl}^{{}} \left[ {\varepsilon_{kl} - \beta_{r} \left( {T_{r} - T_{r0} } \right)\delta_{kl} } \right] + \left( {\alpha_{w} p_{w} + \alpha_{i} p_{i} } \right)\delta_{ij} } \right\}_{,j} + \rho_{e} {\mathop{X}\limits^{\rightharpoonup}}_{i} = 0$$

### Continuity equations

If the unit volume of micro-elements (which can contain representative body elements) is arbitrarily taken at time *t*, and the volume content of each component is Ω, *n*_*r*_、*n*_*w*_、*n*_*i*_ respectively (subscripts *r*, *w*, and *i* represent rock matrix, unfrozen water, and ice respectively, the same below), then according to the law of conservation of mass, the change rate of the volume content of each component during time Δ*t* caused by water migration, ice-water phase transition and thermal expansion and contraction effect is^26^:

5 $$\left\{ \begin{gathered} \dot{n}_{r} = n_{r} \nabla \cdot {\mathop{v}\limits^{\rightharpoonup}} _{w} - \frac{{\rho_{w} - \rho_{i} }}{{\rho_{i} }}n_{r} \dot{n}_{wi} + \beta_{r} \dot{T}n_{r} \hfill \\ \dot{n}_{w} = - \left( {1 - n_{w} } \right)\nabla \cdot {\mathop{v}\limits^{\rightharpoonup}} _{w} - \left( {1 + \frac{{\rho_{w} - \rho_{i} }}{{\rho_{i} }}n_{w} } \right)\dot{n}_{wi} + \beta_{w} \dot{T}n_{w} \hfill \\ \dot{n}_{i} = n_{i} \nabla \cdot {\mathop{v}\limits^{\rightharpoonup}} _{w} + \left( {\frac{{\rho_{w} }}{{\rho_{i} }} - \frac{{\rho_{w} - \rho_{i} }}{{\rho_{i} }}n_{i} } \right)\dot{n}_{wi} + \beta_{i} \dot{T}n_{i} \hfill \\ \end{gathered} \right.$$

where $\dot{n}$ is the change rate of volume content, ${\mathop{v}\limits^{\rightharpoonup}} _{w}$ is the velocity of unfrozen water, *β* is the coefficient of thermal expansion, *T* is the temperature, *ρ* is the density, and *n*_*wi*_ is the volume content of the ice-water phase transition.

By connecting any two formulas in Eq. (5), the expression of the change rate of the volume content of ice-water phase transition can be derived as follows:

6 $$\dot{n}_{wi} = \frac{{\rho_{i} }}{{\rho_{w} }}\left[ {\left( {1 + \frac{{n_{i} }}{{n_{r} }}} \right)\dot{n}_{i} + \frac{{n_{i} }}{{n_{r} }}\dot{n}_{w} + \left( {\beta_{i} - \beta_{w} } \right)\frac{{n_{i} }}{{n_{r} }}n_{w} \dot{T} - \frac{{n_{i} }}{{n_{r} }}\beta_{i} \dot{T}} \right]$$

Within Δt, the volume increment ΔV of the particle Ω due to water migration, ice-water phase transition and thermal expansion and contraction effect can be expressed as:

7 $$\Delta V = \left[ { - \nabla \cdot {\mathop{v}\limits^{\rightharpoonup}} _{w} + \frac{{\rho_{w} - \rho_{i} }}{{\rho_{i} }}\dot{n}_{wi} + \left( {n_{r} \beta_{r} + n_{w} \beta_{w} + n_{i} \beta_{i} } \right)\dot{T}} \right]\Delta t$$

According to the law of conservation of mass of fractured rock mass in cold areas, the continuity equation of rock mass surrounding the tunnel in cold areas can be further derived as^26^:

8 $$\frac{\partial \varepsilon }{{\partial t}} = \mathop {\lim }\limits_{\Delta t \to 0} \frac{\Delta V}{{\Delta t}}{ = } - \nabla \cdot {\mathop{v}\limits^{\rightharpoonup}} _{w} + \frac{{\rho_{w} - \rho_{i} }}{{\rho_{i} }}\dot{n}_{wi} + \left( {n_{r} \beta_{r} + n_{w} \beta_{w} + n_{i} \beta_{i} } \right)\dot{T}$$

Bring Eq. (6) into Eq. (8) to further arrange, we get:

9 $$\begin{aligned} & \frac{\partial \varepsilon }{{\partial t}} + \nabla \cdot {\mathop{v}\limits^{\rightharpoonup}} _{w} - \frac{{\rho_{w} - \rho_{i} }}{{\rho_{w} }}\left[ {\left( {1 + \frac{{n_{i} }}{{n_{r} }}} \right)\dot{n}_{i} + \frac{{n_{i} }}{{n_{r} }}\dot{n}_{w} + \left( {\beta_{i} - \beta_{w} } \right)\frac{{n_{i} }}{{n_{r} }}n_{w} \dot{T} - \frac{{n_{i} }}{{n_{r} }}\beta_{i} \dot{T}} \right] \\ & - \left( {n_{r} \beta_{r} + n_{w} \beta_{w} + n_{i} \beta_{i} } \right)\dot{T} = 0 \\ \end{aligned}$$

### Energy conservation equations

The heat energy transfer modes of rock mass in rock tunnels in cold areas mainly include heat conduction of rock matrix and fluid, heat convection of fissure water, and change of heat energy of fissure rock mass caused by ice-water phase transition. This paper mainly considers the influence of heat convection, heat conduction, and ice-water phase transition on heat energy to establish the energy conservation equation of rock tunnels in cold areas.

10 $$\begin{aligned} & \left( {n_{r} \rho_{r} c_{r} + n_{w} \rho_{w} c_{w} + n_{i} \rho_{i} c_{i} } \right)\frac{\partial T}{{\partial t}} + \left( {n_{r} \rho_{r} c_{r} + n_{i} \rho_{i} c_{i} } \right){\mathop{v}\limits^{\rightharpoonup}} _{s} \cdot \nabla T + n_{w} \rho_{w} c_{w} {\mathop{v}\limits^{\rightharpoonup}} _{w} \cdot \nabla T \\ & = - \nabla \cdot \left[ { - \left( {n_{r} \lambda_{r} + n_{w} \lambda_{w} + n_{i} \lambda_{i} } \right)\nabla T} \right] - \left( {n_{r} \beta_{r} + n_{i} \beta_{i} } \right)T\frac{\partial \varepsilon }{{\partial t}} \\ & \quad - n_{w} T_{w} \frac{{\partial p_{w} }}{{\partial T_{w} }}\nabla \cdot {\mathop{v}\limits^{\rightharpoonup}} _{w} - \nabla \cdot \left[ {\left( {n_{r} \sigma^{\prime}_{r} + n_{i} p_{i} } \right)\frac{{\partial \mathop{u}\limits^{\rightharpoonup} }}{\partial t}} \right] + L\rho_{i} \frac{{\partial n_{wi} }}{\partial t} \\ \end{aligned}$$

where *c* is the specific heat of each component, *T* is the temperature of each component, $\mathop{v}\limits^{\rightharpoonup}$ is the motion speed of each component, *λ* is the heat transfer coefficient of each component, *ε* is the strain of each component, *p*_*w*_ is the pressure borne by liquid water, *p*_*i*_ is the pressure borne by solid ice, $\mathop{u}\limits^{\rightharpoonup}$ is the deformation of each component, and *L* is the latent heat of ice-water phase change.

The governing differential equations for rock tunnels in cold regions, as formed by the three aforementioned equations, are not closed differential equations. When addressing the THM coupling problem within rock tunnels in cold regions, it is imperative to supplement the relationships between various variables, including the stress–strain relationship of fractured rock masses in cold regions, expressions for anisotropic water migration, anisotropic heat transfer, and geometric equations^28^. The finite element method is employed to solve the problem, contingent upon specified initial and boundary value conditions.

## Numerical simulation of the Kunlunshan tunnel

Situated within Qinghai Province along the Qinghai-Tibet Railway, the Kunlunshan tunnel stands as the longest permafrost tunnel globally, boasting a total length of 1686 m. The elevation of the tunnel design is 4600 m with a maximum burial depth of 110 m. The annual average temperature in the tunnel site area is − 4.27 °C, with an extreme minimum temperature of − 37.7 °C. The freezing period lasts for 7–8 months, and the permafrost extends to a depth of 100–110 m. The annual average ground temperature ranges from − 1.81 to − 2.65 °C. The bedrock primarily comprises Triassic slate and sandwiched schist, while the Quaternary strata are predominantly composed of slope deposits and angular gravel. The surrounding rock of the tunnel mainly consists of grade IV and V fractured rocks, which are typical cold region fissure rock masses. The stability of the tunnel is influenced by the coupled effects of Thermo–Hydro–Mechanical field. Therefore, based on the multi-field coupling model established in the preceding section, this section conducts simulations of tunnel construction and operation periods, with a specific focus on investigating the impact of insulation layers on the safety of fractured rock mass tunnels in cold regions.

### Calculation model

**Figure 1:**
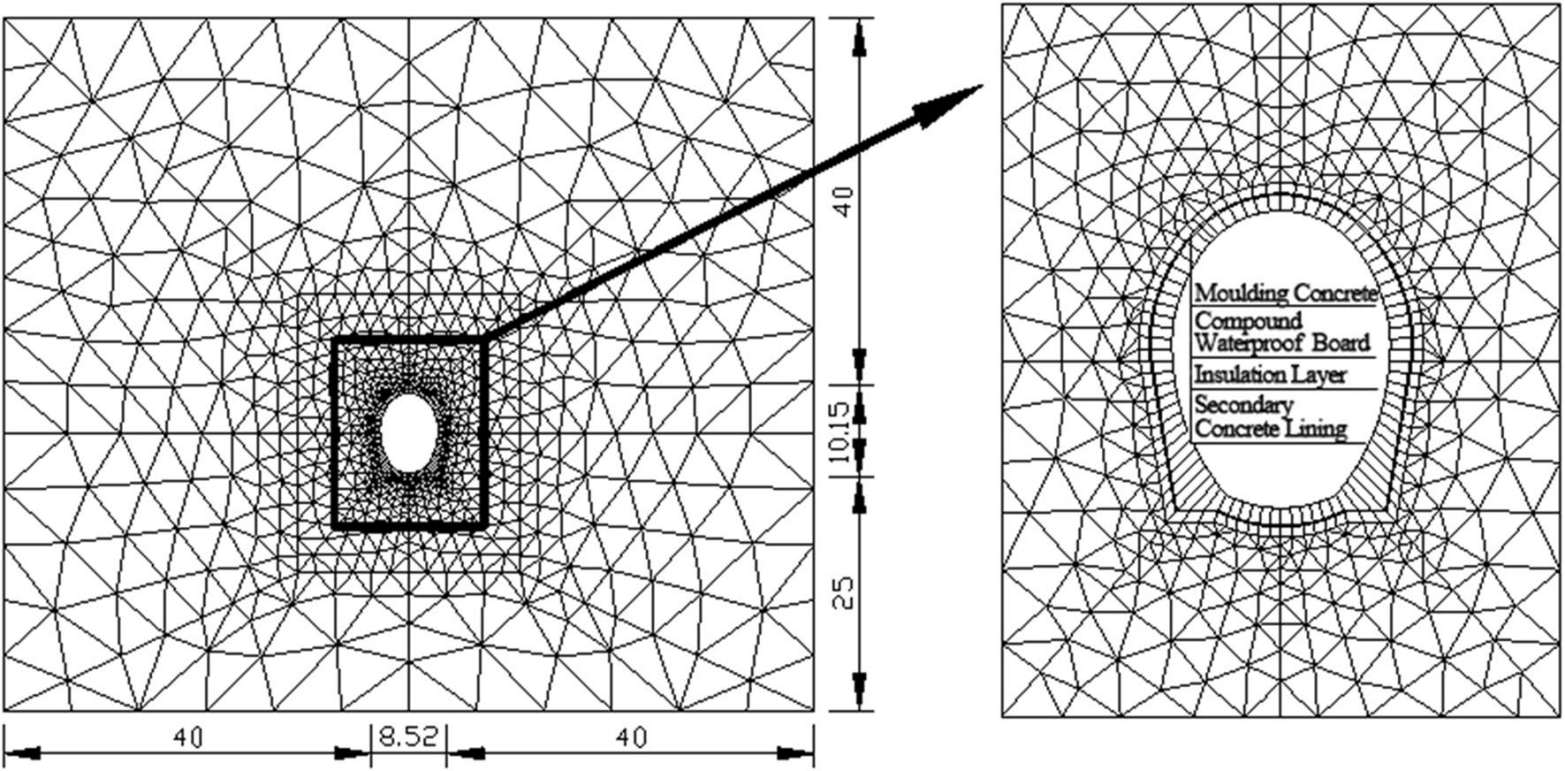
The numerical model.

In accordance with the geological conditions of the Kunlunshan tunnel, cross section DK976 + 410 (Orientation of Bedrock: N67° E/52° N; Joint Attitude N40° ~ 60°E/55° ~ 88°S, N10°W/83°N) at the tunnel entrance is selected as the research section. This cross section has a depth of 40 m and belongs to Grade V surrounding rock. The computational model sets the upper boundary at ground level (40 m), with a bottom rock layer thickness of 25 m and 40 m on both sides. The lateral boundaries encompass a span of 40 m on both the left and right sides. The numerical analysis model is depicted in Fig. 1.

The specific simulation process is as follows: the first step involves simulating the initial conditions, including the temperature field, stress field, deformation field, and moisture field; in the second step, the excavation of the section and construction of primary lining are simulated; then, in the third step, the construction of secondary lining (which occurs one year after excavation and does not bear any construction load) is simulated; finally, in the fourth step, a 10-year operational period is simulated. The primary focus of this simulation analysis lies in examining how a 5 cm-thick insulation layer impacts tunnel safety.

### Calculation parameters

The anisotropic parameters of fractured rock masses are calculated based the research results of Liu et al^26^. By substituting the fracture parameters and the thermodynamic parameters of the intact rock into the correlation equations established by Liu, the numerical program can solve the anisotropy parameters of the fractured rock mass. The pertinent data sources^29,30^ are presented in Table 1.

**Table 1:** The thermal and mechanics parameters of the surrounding rock and support systems.

Parameters	V-class surrounding rock	Concrete	Insulation	Unfrozen water	Ice
Specific heat capacity *C* /(J kg^-1 ^℃^−1^)	850	993	5000	4200	1930
Heat transfer coefficient λ/(W m^−1 ^℃^−1^)	2.5	1.93	0.03	0.54	2.22
Elastic modulus E /GPa	0.90	29.5	14.6	–	–
Poisson's ratio *ν* (thaw/freeze)	0.40/0.35	0.20	0.20	–	–
Cohesion *C* /MPa (thaw/freeze)	0.06/0.50	2.00	–	–	–
Internal friction Angle *φ* /° (thaw/freeze)	45/25	60	–	–	–
Density *ρ* /(kg m^−3^)	2500	2600	600	1000	916.8

The area contact ratio of bedding planes is set to 0.5, and the area contact ratio of dominant joints is set to 0.2. The width of the fractures in the bedding planes is assumed to be 1 mm, while the width of fractures in dominant joints is assumed to be 2 mm. The flexibility tensor, permeability tensor, and equivalent heat transfer tensor of fractured rock mass are automatically calculated by the program. The specific heat capacity, thermal conductivity coefficient, expansion coefficient, and density of fractured rock mass are determined using a weighted average based on mixture theory, with all parameters varying with temperature^31^. The Poisson's ratio, cohesive strength, and internal friction angle have small variations during both freezing and melting conditions. The elastic modulus can be expressed as *E* = 950-20* T* + 1.5*T*^2^, where *T* represents the mixed temperature of fractured rock mass in degrees Celsius (°C). The thermal expansion coefficient for rocks is uniformly taken as 10.8 × 10^–6^ /°C, for ice it is 51 × 10–6 /°C, and for water it is 21 × 10^–6^ /°C.

### Initial-boundary value conditions

Drawing from Xu^29^ and Tang^30^, the temperature atop the Kunlunshan tunnel is represented in trigonometric form (with the initial moment set in early September), as follows:

11 $$T_{a} = - 5.2 + \frac{1.5}{{30 \times 12}}t + 12\sin \left[ {\frac{2\pi }{{12 \times 30}}\left( {t + 336} \right) + \frac{11}{{12}}\pi } \right]$$

In the formula above, *T*_*a*_ represents temperature in degrees Celsius (°C), while t denotes time, measured in months. We consider a temperature increase of 1.5 °C over a span of 30 years.

Based on references from Xu^29^, the average geothermal temperature in the Kunlunshan region is approximately − 2.5 °C. By combining air temperature data with measured geothermal temperature data, we employ a geothermal gradient of 3% to deduce the initial geothermal field. Convective heat transfer between the air and the upper boundary of the model is in effect, with the coefficient of convective heat transfer between the air and the ground being *h* = 15.0W/(m^2^·°C). Notably, the model's lateral boundaries are treated as adiabatic boundaries, while the lower boundary of the model and the inner wall of the tunnel serve as heat flow boundaries, with a heat flux rate of 0.06 W/m^2^. Given the extended construction duration of the tunnel, human activities impact the air temperature within the tunnel during the construction period. We derive the formula for tunnel air temperature during the construction period (lasting one year) through fitting to measured data. This yields the following formula:

12 $$T_{a} = \left\{ \begin{gathered} 14.9\sin \left[ {\frac{2\pi }{{12 \times 30}}\left( {t + 336} \right) + \frac{11}{{12}}\pi } \right],\sin \left[ {\frac{2\pi }{{12 \times 30}}\left( {t + 336} \right) + \frac{11}{{12}}\pi } \right] > 0 \hfill \\ 3.00\sin \left[ {\frac{2\pi }{{12 \times 30}}\left( {t + 336} \right) + \frac{11}{{12}}\pi } \right],\sin \left[ {\frac{2\pi }{{12 \times 30}}\left( {t + 336} \right) + \frac{11}{{12}}\pi } \right] \le 0 \hfill \\ \end{gathered} \right.$$

Based on the geological report, the assumption is made that the water field is saturated, with external water recharge taken into account. The model is configured with impervious boundaries on both sides and at the bottom, while the tunnel wall serves as permeable boundaries. Dealing with the boundary conditions of the deformation field, we establish the following:

1. The lower boundary is subject to a fixed constraint.
2. The boundaries on both sides feature normal constraints.
3. The upper boundary is left free.
4. Taking into consideration the field measurement data, it is important to note that the tectonic stress in this area is relatively low, with primary consideration given to gravity-induced stress.

## Numerical results and analysis

Through the calculation model established in Chapter 2, numerical simulations were conducted on the deformation and stress of the surrounding rock, primary lining, and second lining of the Kunlunshan tunnel at different periods. The calculation results are as follows:

### Deformation and stress of surrounding rock

**Figure 2:**
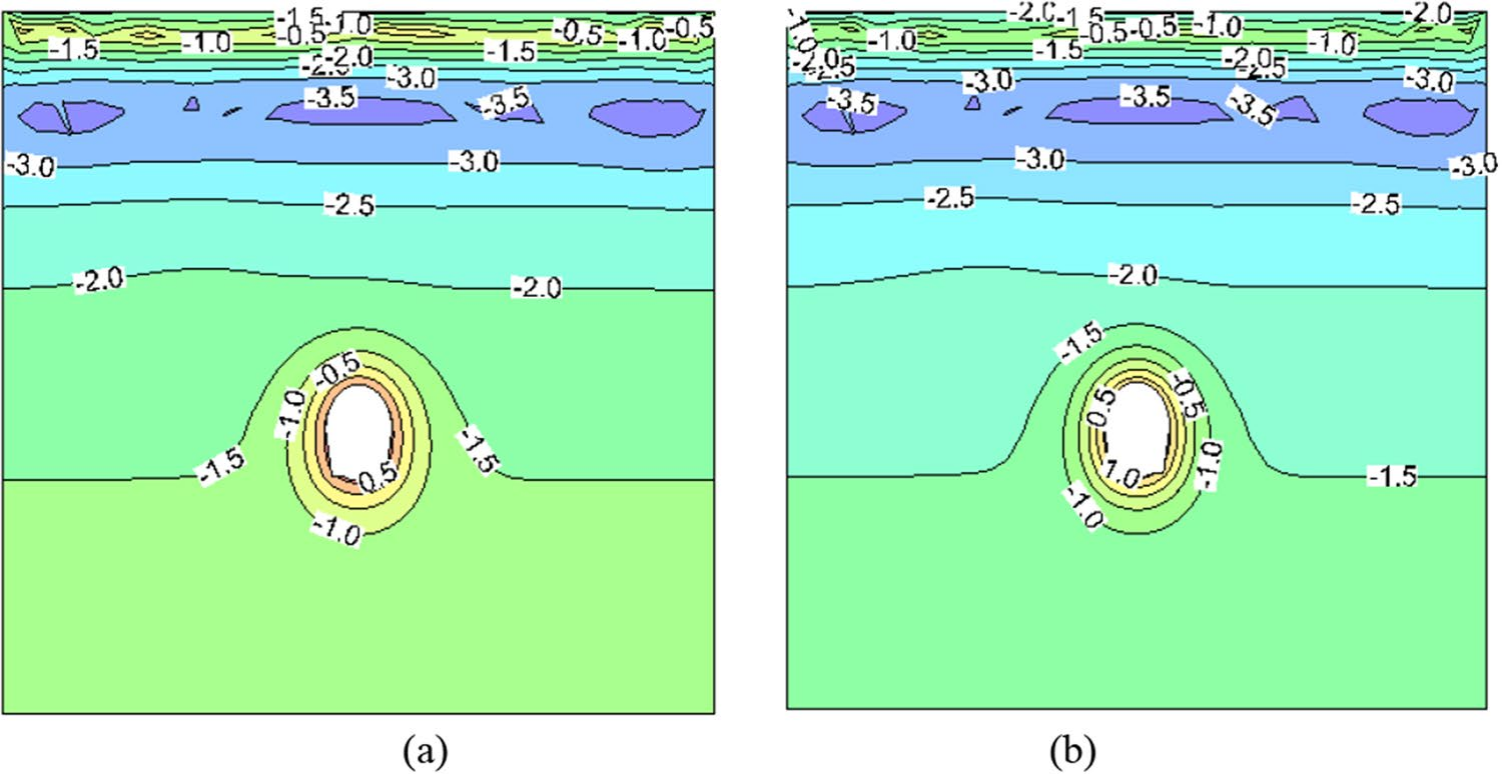
The temperature field of surrounding rock at construction period (unit: ℃). (**a**) No thermal insulation layer. (**b**) 5 cm thermal insulation layer.

**Figure 3:**
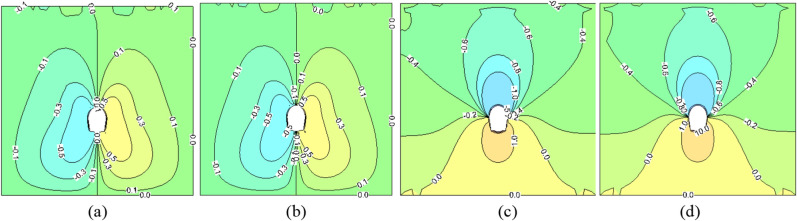
The deformation field of surrounding rock at construction period (unit: mm). (**a**) No insulation (horizontal direction). (**b**) 5 cm insulation (horizontal direction). (**c**) No insulation layer (vertical direction). (**d**) 5 cm insulation layer (vertical direction).

**Figure 4:**
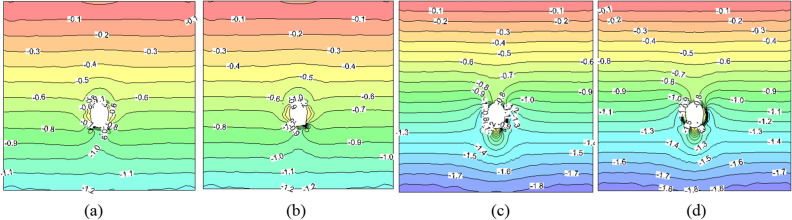
The stress field of surrounding rock at construction period (unit: MPa). (**a**) No insulation (horizontal direction). (**b**) 5 cm insulation (horizontal direction). (**c**) No insulation (vertical direction). (**d**) 5 cm insulation (vertical direction).

Upon the conclusion of construction in October 2002, the temperature field of the surrounding rock under various insulation schemes is depicted in Fig. 2, the deformation field of the surrounding rock is illustrated in Fig. 3, and the stress field of the surrounding rock (with tension represented as positive and pressure as negative) is presented in Fig. 4.

Figure 2 reveals that, owing to the presence of cracks, the temperature field of surrounding rock during construction period is not completely symmetrical. When the construction is completed, the temperature distribution of the surrounding rock in both scenarios exhibits a consistent pattern, forming a circular isotherm around the tunnel. By comparing Fig. 2(a) and (b), it can be observed that the presence of an insulation layer reduces the depth of melting in the surrounding rock of the tunnel. With a 5 cm insulation material installed, the maximum depths of melting for vault, sidewall, and arch bottom are measured to be 1.84 m, 3.26 m, and 2.59 m respectively. According to Xu's data^29^, based on isotropic theory, these depths should theoretically reach values of 2.19 m, 3.41 m, and 2.83 m respectively. However, the measured melting depths were 1.8 m, 3.5 m, and 2.5 m respectively. Clearly, the simulated melting depths obtained in this study are more consistent with the measured values.

From Fig. 3, it can be observed that both horizontal and vertical deformations occur only within a small range around the cavity, with significant asymmetry in vertical deformation between the two proposals. When a 5 cm insulation layer is installed, the deformations of the vault, sidewall, and arch bottom towards the cavity are measured at 9.01 mm, 36.90 mm, and 13.60 mm respectively; without an insulation layer, these values are measured at 9.15 mm, 36.94 mm, and 14.01 mm respectively. It is evident that due to the relatively short duration of insulation layer construction time, its impact on surrounding rock deformation is not significant by the end of construction.

Figure 4 depicts the occurrence of stress concentration and redistribution in the vicinity of the tunnel following excavation, marked by noticeable asymmetry. The maximum horizontal stress surrounding the tunnel measures approximately − 0.55 MPa and − 0.61 MPa, respectively, with and without the presence of a 5 cm insulation layer. Concurrently, the maximum vertical stress registers at approximately − 0.70 MPa. Stress levels align with corresponding deformation within the surrounding rock, and the impact of the thermal insulation layer on surrounding rock stress during the construction period remains inconspicuous. Due to the fact that the lining of this model does not bear any construction load and the presence of cracks enhances the deformation capacity of surrounding rocks, stress release and adjustment are more thorough. Consequently, in this study, the deformation value of surrounding rocks exceeds Xu's^29^ calculated result (7.23 mm), while the stress value is lower than their findings (− 2.89 MPa).

### Stress situation of the primary lining

**Figure 5:**
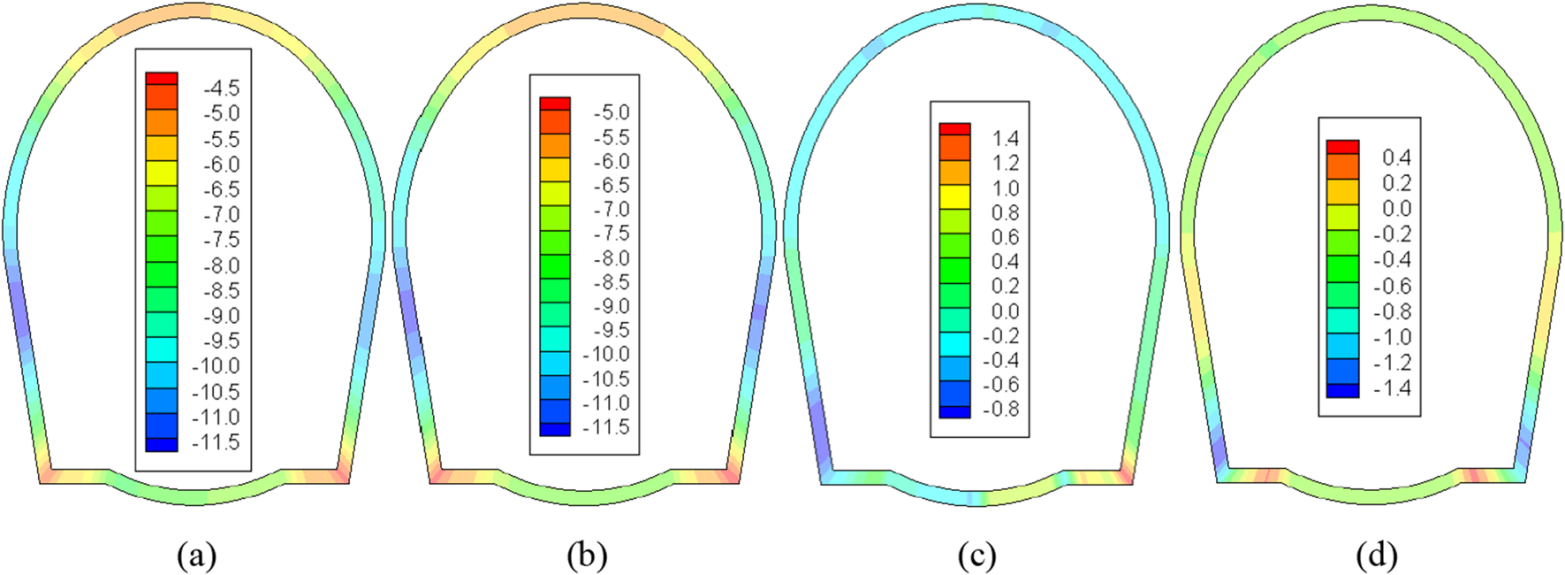
The stress field of the primary lining after a construction period (unit: MPa). (**a**) No insulation layer (*σ*_max_). (**b**) 5 cm insulation layer (*σ*_max_). (**c**) No insulation layer (*σ*_min_). (**d**) 5 cm insulation layer (*σ*_min_).

As observed in Fig. 5, the primary lining structure bears a significant portion of the load and frost heave pressure during construction, leading to substantial stress under both scenarios. In the absence of an insulation layer, the maximum compressive stress emerges at the midpoint of the left sidewall, measuring approximately 10.02 MPa. Conversely, the maximum tensile stress is situated at the right arch foot, amounting to approximately 1.40 MPa. Upon the inclusion of a 5 cm insulation layer, the maximum compressive stress is similarly located at the midpoint of the left sidewall, at around 9.70 MPa. The tensile stress region diminishes significantly, with a maximum value of approximately 0.4 MPa recorded near the invert, roughly 2 m away from the arch foot. Evidently, the thermal insulation layer notably enhances the stress within the supporting structure, and the absence of such insulation may jeopardize the integrity of the primary lining. Furthermore, as the primary lining shoulders a greater load during the construction phase, its stress levels surpass those obtained by Xu^29^ (-9.25 MPa).

**Figure 6:**
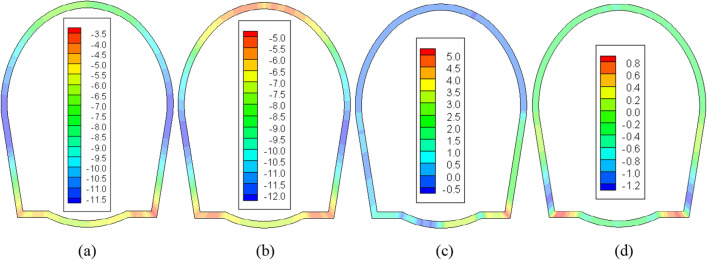
The stress field of the primary lining after operating for 10 years (unit: MPa). (**a**) No insulation layer (*σ*_max_). (**b**) 5 cm insulation layer (*σ*_max_). (**c**) No insulation layer (*σ*_min_). (**d**) 5 cm insulation layer (*σ*_min_).

As the rock surrounding the tunnel reverts to a frozen state after a decade of operation, the primary lining structure must contend with substantial frost heave pressures, evident in Fig. 6. Consequently, its stress experiences a significant upswing compared to the construction phase. Without an insulation layer, the maximum compressive stress rises to 11.60 MPa, the maximum tensile stress escalates to 4.50 MPa, and the extent of the tensile stress area markedly expands. When a 5 cm insulation layer is introduced, the maximum compressive stress elevates to 11.66 MPa, and the maximum tensile stress increases to 0.80 MPa, with a smaller tensile stress region compared to the scenario without insulation. Absent an insulation layer, the lining is at risk of damage due to excessive tensile stress. Additionally, as per reference 29, the maximum tensile stress of the primary lining after 30 years of operation is calculated to be 3.45 MPa, and the maximum compressive stress is 11.72 MPa, both of which are higher than the simulated values in this paper. This discrepancy can primarily be attributed to reference 29's consideration of a larger construction period load on the primary lining.

### Stress situation of the second lining

**Figure 7:**
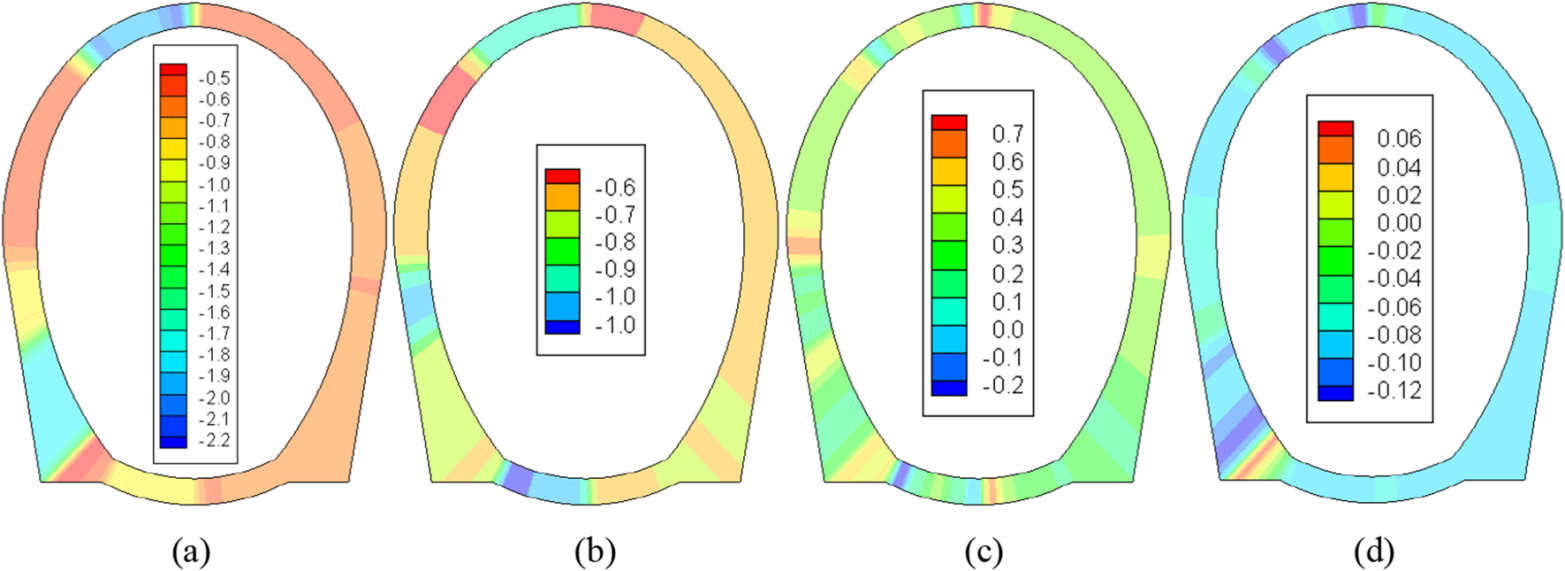
The stress field of the second lining after a construction period (unit: MPa). (**a**) No insulation layer (*σ*_max_). (**b**) 5 cm insulation layer (*σ*_max_). (**c**) No insulation layer (*σ*_min_). (**d**) 5 cm insulation layer (*σ*_min_).

**Figure 8:**
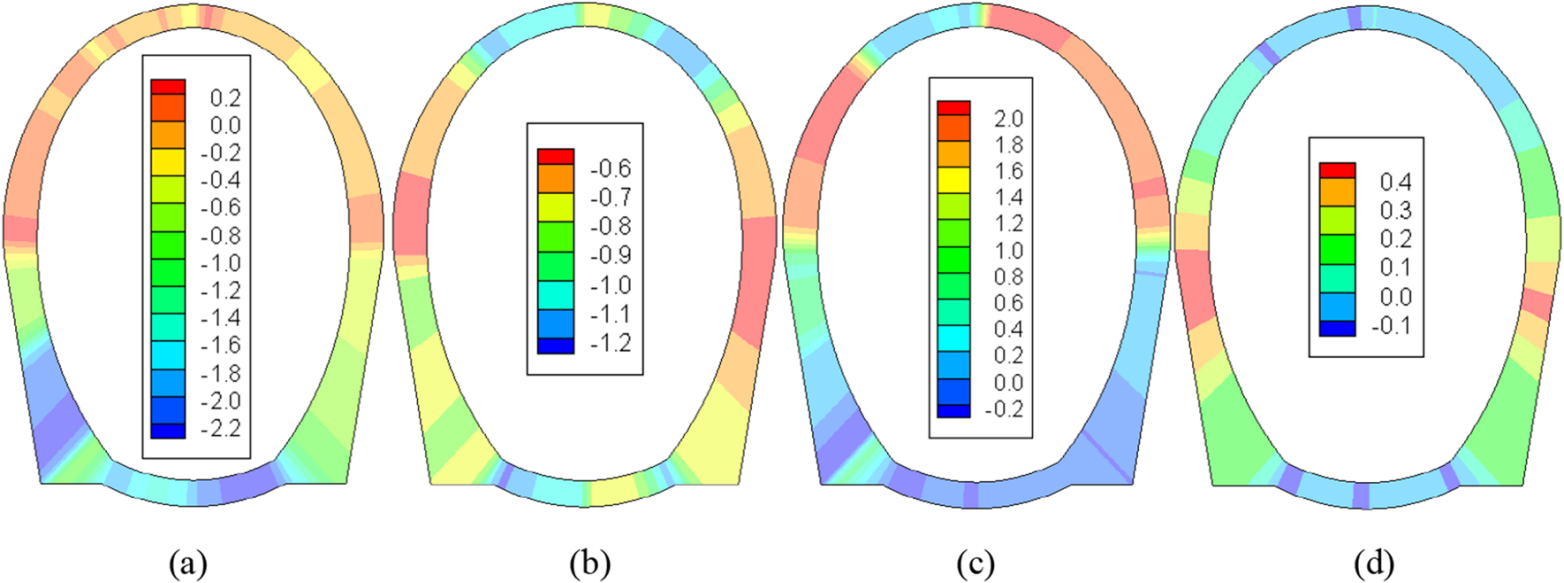
The stress field of the second lining after operating for 10 years (unit: MPa). (**a**) No insulation layer (*σ*_max_). (**b**) 5 cm insulation layer (*σ*_max_). (**c**) No insulation layer (*σ*_min_). (**d**) 5 cm insulation layer (*σ*_min_).

The stress situation of the second lining structure during the construction period and after 10 years of operation when laying a 5 cm insulation layer and without insulation layer are shown in Figs. 7 and 8.

Considering that the stress release within the surrounding rock is substantially accomplished by the time the second lining is implemented, one year following the tunnel excavation's conclusion, the numerical simulation does not impose a load on the secondary lining during the construction phase. Consequently, the stress levels within the second lining structure, as depicted in Fig. 7, are notably lower than those computed in reference 29(with a maximum compressive stress of 3.53 MPa and a maximum tensile stress of 1.31 MPa). Moreover, the distribution displays greater asymmetry compared to that of the primary lining. In the absence of an insulation layer, the maximum stress within the second lining is positioned atop the left arch, measuring approximately 2.20 MPa. Conversely, the maximum tensile stress occurs at the left arch foot, at around 0.75 MPa. Introducing a 5 cm insulation layer results in a maximum compressive stress of approximately 1.42 MPa within the second lining, located at the arch foot's interior. Meanwhile, the maximum tensile stress decreases significantly to 0.06 MPa, emerging within the midpoint of both the arch and the invert. The internal forces on the second lining are primarily caused by frost heave.

Figure 8 illustrates that, owing to the anisotropic THM characteristics induced by the presence of cracks within the rock surrounding the tunnel, the entire section of the second lining is subject to uneven frost heave pressure, leading to a visibly asymmetric stress distribution. After a decade of operation, the large principal stress experiences a slight increase, while the small principal stress undergoes significant changes, with a shift in the location of the extreme value. In the absence of an insulation layer, the maximum tensile stress measures approximately 2.00 MPa, situated on the right side of the arch and the left arch shoulder. Conversely, with a 5 cm insulation layer, the maximum tensile stress is positioned in the middle of both side walls, with a value of roughly 0.45 MPa. This disparity primarily arises from the model's failure to account for the anisotropy of the surrounding rock and the fact that the secondary lining bears 40% of the load during the construction phase. Through comparison, it becomes evident that the absence of an insulation layer would lead to the second lining sustaining damage from tension after a decade of operation. Additionally, Xu et al.^29^ pointed out that in October of the 30th year after the laying of insulation layer, with the temperature rising by 1.5 °C, the surrounding rock would still show a melting circle of about 0.35 m thick under the influence of climate warming, indicating that the tunnel freeze–thaw is sensitive to temperature changes. However, the insulation layer can still play a good role in heat insulation and effectively weaken the frost heave and melt subsidence of surrounding rock. This understanding proves the correctness of the THM model in this paper.

Through the above analysis, it can be seen that the key of THM coupling in rock tunnel in cold region is to determine the calculation parameters accurately. The follow-up research should strengthen the study of the THM coupling parameters of fractured rock mass to make the numerical simulation results more accurate. Furthermore, the study of temperature control measures should be strengthened based on the numerical simulation results to reduce the freezing damage of rock tunnels in cold regions.

## Conclusions

1. The THM coupling model of rock tunnel in cold regions is established. The model can reflect the anisotropy of deformation, water migration and heat transfer of fractured rock mass caused by initial fissure in rock mass.
2. The construction and operation process of Kunlunshan tunnel are simulated based on the THM model. The thermal insulation layer has little influence on the deformation of surrounding rock and the stress of supporting structures during construction period, but has significant effect during operation period.
3. The established THM model can reflect the anisotropy characteristics caused by fractures. Both the temperature field of surrounding rock and the stress distribution of supporting structure show obvious asymmetry.
4. To improve the accuracy of numerical simulation of rock tunnel in cold regions, the research on the THM coupling parameters should be strengthened.

## Data Availability

Data analyzed in this study are available upon reasonable request from the corresponding author (Naifei Liu).
